# Exploring nurses’ experiences transitioning from clinicians to professors at Ontario colleges

**DOI:** 10.1177/08445621251320708

**Published:** 2025-04-16

**Authors:** Michelle Greenway, Emily Belita, Pamela Baxter, Joanna Pierazzo, Sheila Boamah

**Affiliations:** 1 Student 62703 McMaster University;; 2 62703 McMaster University; 3 62703 McMaster University; 4 62703 McMaster University; 5 62703 McMaster University

**Keywords:** nursing education, Ontario colleges, transition, nursing faculty, professor

## Abstract

**Background:**

In 2022, Ontario colleges and universities reported an estimated 67 vacant full-time nursing faculty positions, driving significant recruitment of nurses directly from clinical practice. Many of these nurses transition to academia lacking the necessary pedagogical preparation and socialization for a faculty role, leading to feelings of inadequacy, stress and an increased intent to leave their positions.

**Objective:**

This qualitative descriptive study explored nurses’ experiences as they transitioned into the professor role to identify strategies to decrease transition stress, improve career satisfaction, and decrease early-career nursing faculty attrition at Ontario colleges.

**Methods:**

Data were collected in semi-structured interviews with nine participants from Ontario colleges offering the Bachelor of Science in Nursing degree and analyzed using Conventional Content Analysis.

**Results:**

Study findings detailed their emotional experiences, diverse preparations before becoming a professor, and the challenges navigating their new role. The study provided three major themes: 1) emotional aspects of the transition experience, 2) preparation for the nursing professor role, and 3) navigating the role and college setting. Nursing professors desired improved orientation programs, formal mentorship opportunities and socialization to the nursing professor role.

**Conclusion:**

The findings underscore the need for evidence-informed orientation programs that provide comprehensive training in institutional policies, nursing pedagogy, and support in adapting to the academic culture. These findings can guide Ontario colleges in offering standardized orientation programs that support nurses’ excelling as professors and improve retention of this important group.

## Background and Purpose

The global nursing shortage extends to nurse faculty positions, with a critical shortage across North America (World Health Organization, 2020). Canada reports one of the most significant vacancies to date among countries in this region. The Canadian Association of Schools of Nursing projected 425 vacancies in full-time permanent and long-term contract nursing faculty positions in Canada for 2023 (Canadian Association of Schools of Nursing [CASN], 2023). There are multiple factors contributing to the nursing faculty shortage in Canada. These include a lack of qualified nursing faculty, heavy academic workloads, and reported low job satisfaction among faculty members (Boamah et al., 2021; CASN, 2023). Additionally, the dynamic landscape of nursing and nursing education during the COVID-19 pandemic caused increased stress and burnout for nursing professors, leading to decreased intent to stay in their roles (Boamah, 2022; Kissel et al., 2023; Labrague & de los Santos, 2021). Furthermore, the nursing faculty shortage continues to be exaggerated by retirement, as exhibited by the departure of 91 permanent nursing faculty members across Canada in 2022 (CASN, 2023; Yedidia, 2014).

As academic institutions recruit nurses from clinical practice to fill faculty positions, it is becoming evident that these nurses often need additional preparation in pedagogy to effectively transition to the academic setting (Smith et al., 2019). The lack of required training and socialization for a faculty role can leave new recruits feeling insecure, stressed, and anxious, ultimately impacting the quality of their teaching (Summers, 2017; Suplee et al., 2014). The novice period (denoting less than three years of full-time experience in the role) is particularly precarious, as shown in a mixed-method study by Jeffers and Mariani (2017), where 52% of nursing faculty members reported an intent to leave academia early in their careers. Calaguas (2023) alluded to the fact that this may be because the transition from clinician to faculty member is akin to the experience of a newly graduated nurse transitioning to clinical practice, often described as a “transition shock” (Duchscher, 2009).

Transition shock is defined as the most immediate and dramatic stage in professional role adaption (Duchscher, 2009). It is described as *jumping into the deep end* and can occur due to the contrast between the knowledge, responsibilities, and expectations of a familiar role with that of a new and unfamiliar one (Duchscher, 2009, p.1105). A major contributor to transition shock is inadequate role preparation as many novice professors feel unprepared for their transition (Brower et al., 2022; Shapiro, 2018; Wendler et al., 2021). Several strategies have been proposed in the nursing literature to aid in healthy and successful professional transitions of nursing faculty. These include mentorship, onboarding, and socialization within the academic community (Brower et al., 2022; Fritz, 2018; Grassley et al., 2020; McPherson & Wendler, 2023; Nicholls & Kelman, 2023; Summers, 2017). Specifically, structured mentorship programs have been shown to facilitate the transition to faculty roles for nurses, but they are often missing from School of Nursing orientations (Fang et al., 2016; Fritz, 2018; Nowell et al., 2017; Vandyk et al., 2017).

While various international studies (i.e., Australia, Ireland, Thailand, United Kingdom, United States) have explored the transition experience of nurses as they move from clinician to professor, there is a notable gap in the literature when it comes to the perspectives of Ontario college faculty (Boamah et al., 2021; Fritz, 2018; Grassley et al., 2020; Schoening, 2013). The Ontario government has promised to increase Bachelor of Science in Nursing (BScN) graduates to address the nursing shortage and to increase the province’s healthcare capacity (Rushowy, 2021). One strategy released was a 2020 policy allowing colleges to offer nursing degrees independently (stand-alone) from a university, increasing the demand for nursing faculty members in the province (Government of Ontario, 2020). As the demand for nurses and nursing professors in Ontario continues to rise, it becomes crucial for nursing schools in Ontario colleges to gain insight into the experiences of novice nursing professors to improve recruitment and retention (CASN, 2023; Rushowy, 2021). This study aimed to address this gap by exploring two key research questions: *What are the experiences of registered nurses when transitioning from clinicians to professors at Ontario colleges, and what factors influence these experiences?*

The insights gained from this research can enhance the understanding of the experiences of nursing faculty members and inform strategies to reduce transition shock, ultimately contributing to increased job satisfaction and higher retention rates.

## Methods and Procedures

### Study Design

In this study, a qualitative descriptive design was deemed appropriate because of the subjective nature of participants’ experiences and the unknown perspectives of nursing college professors (Neergaard et al., 2009; Sandelowski, 2000).

### Setting and Sample

Participants were recruited from Ontario colleges offering a BScN degree. Ontario is Canada’s most populated province and second largest by area. Based on the predicted information power of the data and previous qualitative research on nursing faculty, an initial sample size of 11-20 participants was targeted to explore the research questions (Grassley et al., 2020; Malterud et al., 2016; Patton, 2002). However, fewer participants were necessary to reach data saturation, so recruitment stopped after nine participants. Participants were recruited based on the following inclusion criteria: 1) employed full-time as a nursing faculty member at an Ontario college, 2) teaching in a BScN degree program, and 3) started a full-time teaching position in the past three years. The transition experiences of nursing faculty teaching in an academic setting were the focus of the study and, therefore, faculty members who were exclusively teaching in the clinical setting, or held part-time or casual positions were not included due to the differences in the responsibilities of the roles (CASN, 2022). Participants were recruited using three methods: (a) indirectly through the deans and directors of Ontario colleges, (b) through advertisements on social media (e.g., Twitter and LinkedIn), and (c) through snowball sampling (word-of-mouth). Once initial participants were recruited through purposeful criterion sampling, snowball sampling was used to recruit additional participants (Lockard, 2016; Moser & Korstjens, 2018). Participants were asked to forward the researchers information and recruitment poster to other qualifying participants in their social or professional contacts ***.***

### Data Collection Procedures

The primary researcher conducted semi-structured interviews over videoconference (Zoom for Education), as participants were located across the province. One audio interview and eight audio-video interviews were completed, each lasting approximately one hour. All interviews occurred at an agreed-upon and participant-preferred time. The researcher took handwritten notes of any physical cues noted during the virtual interview but due to the high identifiability of video, only the audio was recorded. An interview guide consisting of ten questions was used to guide the interviewing process. The interview guide was informed by Transitions Theory (Meleis, 2010) to explore the personal, social and community conditions that influenced their career change and transition experience. All participants were provided with an honorarium of $15 for their time.

### Data Analysis Procedures

The interviews were audio recorded through Zoom and transcribed using Trint transcription software then reviewed, corrected and de-identified by the primary researcher (https://trint.com, 2023). De-identified transcripts were stored and organized using NVivo qualitative software (NVivo14, Copyright © 1999-2023). Initial data analysis, using conventional content analysis, began after the first interview to ensure any potential and unexpected themes could be explored in subsequent interviews (Chhokar, 2023; Moser & Korstjens, 2018). The primary researcher independently coded and then collaboratively reviewed the codes iteratively with the research team. The research team was comprised of experts in qualitative research methods and the subject of nurse faculty experiences. After establishing an agreed-upon coding scheme, the codes were then sorted into categories and meaningfully grouped into themes with consideration of the personal, social and community conditions impacting their transition (Elo & Kyngäs, 2008; Hsieh & Shannon, 2005, Meleis, 2010).

## Results

Nine nursing faculty members from four Ontario colleges who were in their first three years of full-time teaching in a BScN program participated. The four colleges represented northern, eastern, and southwestern Ontario, and included both urban and rural settings. All participants identified as women. At the time of the interviews, the participants’ ages ranged from 20-60 years old, with six participants falling within the 40-50 years age range. Regarding the highest completed level of education, seven participants held master’s degrees, one had completed a Ph.D., and one participant was currently enrolled in a Ph.D. program.

The findings of this study were categorized into three major themes: 1) the emotional aspects of the transition, 2) preparation for the nursing professor role, and 3) navigating the role and college setting (Table 1).

**Table 1.** Major themes and subthemes.

Theme 1: Emotional aspects of the transition experience	Theme 2: Preparation for the nursing professor role	Theme 3: Navigating the role and college setting
**Negative Emotions** • Overwhelmed and anxious • Surprised or Transition shock • Feeling alone	**Formal Preparation** • Graduate education • Orientation programs	**Formal Support** • College faculty development programs • Mentorship
**Positive Emotions** • Contentment with work-life balance • Gratitude • Feeling valued	**Informal Preparation** • Clinical experience	**Informal Support** • Socialization

### Theme 1: Emotional Aspects of the Transition Experience

The first theme, “Emotional aspects of the transition experience” reflects both the negative and positive emotions participants experienced as they transitioned from clinician to a full-time professor role. There were three subthemes describing the negative emotions they experienced: 1) feeling overwhelmed and anxious, 2) feeling surprised and transition shock, 3) feeling alone; and three subthemes describing their positive emotions, 1) contentment with work-life balance, 2) gratitude, and 3) feeling valued.

#### Overwhelmed and Anxious

The majority of the participants described how the beginning of the semester or school year was especially difficult as there was a lot of material to become familiar with in a short period of time. Four of the participants spoke about how they felt overwhelmed by the steep learning curve. One participant shared,

*I think the only part that was kind of shocking was that the fall course load is very heavy, especially for a person who just started. It was just way more than I had expected in regard to preparation because I had to learn a bunch of things all at once. So not just teaching for the first time, three new courses plus the system, because it was a different learning management system that I was used to.* (Participant 1)

In addition to feeling overwhelmed, one participant described a feeling of “separation anxiety.” She mentioned that although she was an expert in patient care and patient education at the bedside, she felt anxious and uncertain about dealing with a new “population” (nursing students). She shared, *“It's that little bit of that separation anxiety from clinical and not feeling confident. You can’t start at the finish line.”* (Participant 2)

Although holding multiple positions was a contributing factor to their stress and heavy workload, all study participants maintained a clinical position in addition to their faculty role. There were various motivations for remaining in the clinical setting including financial supplementation, staying current with clinical skills, and sustaining relationships with clinical co-workers. Despite the participants' descriptions of feeling overwhelmed with their new responsibilities as faculty members, there was comfort and security in maintaining their clinical roles.

#### Surprised or Transition Shock

In seven out of the nine interviews, participants expressed surprise at the preparation needed for each course and the amount of administration work outside of teaching. One participant expressed her vulnerability by describing how she was shocked by the responsibilities of the role, stating:

*I was coming from a situation in bedside nursing where you feel competent, and you feel secure of your skills, and you know the policies and you know the rules and the guidelines. So as a result of that, it was very eye opening and humbling.* (Participant 2)

Another surprise conveyed by participants was the amount of emotional support professors provided to students beyond class hours. According to the participants, many students experienced mental health issues and faced their own stresses due to the transition to post-secondary education. Participants disclosed the considerable time they spent offering emotional support and counselling to their students, which they were not expecting:

*Some of them have mental health issues with anxiety or depression. It's too overwhelming for them. And so, they'll come and talk. I did not expect to be a counsellor.* (Participant 3)

#### Alone

Another emotion experienced by eight of the nine participants was the feeling of being alone. One participant spoke about working in “silos,” describing how they felt cut off from other faculty members, especially during the height of the pandemic. They were used to a team-based approach to nursing where they could discuss a complex case or ask for advice. She shared:

*So, I'm coming from settings where we were very heavily continually communicating, problem-solving, debriefing daily, almost patient specific. To now, I do find that working as a faculty is very siloed and you can be very isolated.* (Participant 4)

Additionally, six of the participants felt alone in learning the online platforms and administrative tasks needed for the faculty role. The COVID-19 pandemic moved most of the training to online asynchronous modules, potentially exacerbating their feelings of isolation. This led to participants feeling as though they wasted a lot of time learning technology and administration by trial and error.

*It's just such a steep learning curve to learn the IT aspect. I really wish that we had an assigned individual that would work through (it with us). But it took me so much time to figure that out first semester.* (Participant 4)

Although the participants expressed negative emotions related to their transition experiences, they also highlighted the many positive emotions they experienced as they moved into the professor role. For many, being a faculty member was a career goal, and they were excited to teach and give back to the nursing profession.

#### Contentment with Work-life Balance

A positive aspect of the transition reported by participants was the flexibility and work-life balance associated with the professor role as compared to shift work they had in the hospital setting. One nurse noted how she had flexibility in her day and her workplace. She was often able to work at home after her child went to bed and, therefore, felt better about being present for her family. Another participant shared: *It is work life balance because you are off for two months and that is huge. I am tired of shift work. I cannot do that anymore. The hospital is brutal.* (Participant 1)

Similarly, four participants shared how the college setting was more positive compared to the hospital setting. The participants expressed that the current hospital culture is incredibly stressful and self-sacrificial in a post COVID-19 era. One participant shared: *Shift work was taxing. The stress from the pandemic. Everything was so sacrificial in the nursing world. And I love the (clinical) population. But it was coming at a cost.* (Participant 2)

Overall, the participants spoke positively of the flexibility of the work hours in a faculty position. They shared that although there were busy periods as professors, they still felt content with the work-life balance compared to working clinical shifts at an acute care hospital.

#### Gratitude

Gratitude was another common positive emotion revealed by the participants. All the participants felt grateful for their jobs and the opportunity to be a nursing professor. Six of the participants expressed gratitude for academic career opportunities, such as building electives, writing policy and updating the curriculum. They were enthusiastic and passionate about teaching nursing as noted by this participant who stated,

*You get a sense of contribution, and especially if you are that type of nurse who enjoys that sense of development and project development and contributing to nursing education, whatever it might be, there's a lot of opportunity in the institution.* (Participant 5)

#### Feeling Valued

Finally, making a positive impact on nursing students’ experiences was fulfilling for all participants. One participant described how positive feedback from her students helped build confidence and purpose in her work as a nursing professor. Similarly, one participant felt valued and welcomed in her college community and stated that this decreased her stress:

*I think that it's such a great place to learn. So, I feel valued. If anything, I think that wherever hires you, they need to show that they value you.* (Participant 1)

Each participants expressed a combination of negative and positive emotions during the interviews. The participants consistently stated the emotions they experienced throughout their transitions were influenced by their preparation and ability to navigate their roles.

### Theme 2: Preparation for the nursing professor role

The second theme describes the academic and professional preparations needed before the role begins. Subthemes include: 1) education, 2) orientation programs, and 3) transferable clinical skills

#### Education

Five of the nine participants shared that their graduate education lacked opportunities for practical teaching experience, and that this significantly influenced their level of teaching preparedness. For instance, one participant who focused on research for her graduate work admitted to having no formal preparation for teaching. She shared:

*Formal teaching on teaching? No. I can't think of really any time, even self-education. I kind of just dove right in. Started teaching without any sort of learning how.* (Participant 6)

Most of the participants felt unprepared to teach post-secondary education after completing their graduate education because of limitations within the existing curriculum and lack of opportunities for practical application.

#### Orientation Programs

Participants noted a general lack of orientation programs provided to them before beginning their positions. One participant voiced her frustration with the lack of orientation at her college, specifically in the nursing school:

*The other piece of that, I would say for our program in particular, without being too specific, there wasn't a lot of orientation support to the full-time role, which I would assume is a bit of a theme. There's not a go to, there's not standard policy or procedures, there's no this is where you find things. So, there are lots of unanswered questions at start-up.* (Participant 5)

A major gap in the development programs was an orientation to the services available to students and how to navigate student mental health concerns. Many faculty were again, left to figure it out on their own and felt they were doing a disservice to their students by not being properly prepared to help them. One participant shared:

*But when it comes to things like mental health support, when it comes to library support, those weren't conversations that I was given a list of. They're kind of hidden in a website of webs of, you know, you might just fall upon it one day when you're looking for something else.* (Participant 7)

Overall, the participants wished that there were better onboarding processes at their institutions, particularly one that was focused on the BScN program.

#### Transferable Clinical Skills

The participants realized they already had several transferable skills from their clinical settings that helped them in their new roles such as time management, communication, and interprofessional collaboration. One participant spoke of how her organizational skills as a nurse benefited her as a nursing professor,

*Being a nurse, you have to prioritize, you have to be able to organize. You have to be able to facilitate multi-tasking strategies. There's just no denying that. And I think in this role where there is nobody telling you what you have to do every hour you have to prioritize where you start your work.* (Participant 2)

A few participants also had clinical teaching experiences they enjoyed and that developed their pedagogical abilities. One participant shared that as a senior staff member, she often mentored new nurses in her department, which served as informal preparation for academic teaching. Another described her role as a clinical educator, which helped her become comfortable with teaching and understanding faculty expectations.

### Theme 3: Navigating the role and college setting

The final theme encompasses the formal and informal support provided to the participants during their transitions and explores how their socialization to the nursing professor role impacted their experiences. Subthemes include 1) development programs, 2) formal mentorship, and 3) socialization.

#### Development Programs

Most of the colleges included in the study had an ongoing college faculty development program or similar. They had various names for the programs, but all aimed to provide teaching support to college faculty members. The development programs were not specific to the nursing program and included modules that must be completed over the first two years as a full-time faculty member. One participant shared how they were unsure which modules to pick and that it was very self-directed,

*They give you a list and then you go through the list. So, you have to do 12 professional development courses (PD) over a year or two. I would have maybe focused more of my PD courses on how to support students because I feel like that's what I ran into more.* (Participant 8)

Overall, the participants found the programs very helpful in their transition, but some stated it took effort to find the time to complete the modules and that many modules were not applicable to the nursing program. Two participants spoke of how the BScN program is designed to prepare students for the National Council Licensing Exam (NCLEX) and has different goals than many other college programs. Although the development programs were focused on improving adult teaching skills and best practices in pedagogy, they were inadequate to prepare the participants for teaching within the BScN degree programs.

#### Mentorship

The lack of formal mentorship in the BScN program was a common theme. All nine participants identified gaps in understanding the college policies, the service requirements for their role, or how to begin independent research. In these situations, participants yearned for formal mentoring and wished it were provided through their colleges. For example, one participant expressed her desire for a mentor in research,

*It's like, please, please, please, somebody tell me what direction to go. I'd love somebody's advice. I wish that I had that older, wiser individual who could answer that.* (Participant 6)

There was a consensus that formal mentorship should be built into both the mentees’ and mentors’ weekly schedules. The participants did not want to burden their mentors and were conscious of adding to their workload. One participant expressed that formal mentorship training would also be valuable as there were no standards or expectations about the relationship. This led to a mismatch in how much time each person wanted to dedicate to the mentorship relationship, as she needed more support than her mentor expected. Similarly, one participant shared that although she was assigned a formal mentor, it felt informal because there were no expectations of the relationship. She explained: *It was honestly, oh, well, we're going to send you, with her and you're going to mentor her, but I just kind of followed her around. There was no real formal training for that whatsoever, just shadowing.* (Participant 2)

Without clear expectations and goals of the mentorship relationship, novice faculty often searched out other avenues of support. Informal support systems such as senior colleagues, coordinators, and peers (novice nursing faculty) frequently filled the gaps in knowledge during the transition process.

#### Socialization

Socialization or their ability to build relationships and be accepted into academia were major factors in participants’ transition experiences. When the participants felt supported and part of the group, they used more positive terms such as welcoming, collaborative, and camaraderie. Socialization explores all the intangible cultural norms required for relationship building in a new environment and facilitates acceptance by a new group. One participant shared how her team positively impacted her transition,

*But the group of people can make or break it. Everybody I have worked with has been pretty darn amazing. So that's been helpful.* (Participant 2)

Being a part of the team was also described as camaraderie in the interviews. A few of the participants had the opportunity to co-teach a course with a more experienced faculty member. One participant shared how co-teaching allowed her to build relationships and helped her join the community,

*So just that camaraderie, which I think the co-teaching thing was great for. I feel like I really started to get into the community with that and the teacher that I taught with we drove to work together. She showed me around campus. When other people came up and talked to her, I would talk to them as well. I started to make connections. (Co-teaching) was just a really great experience, and it made me feel a lot more immersed in the community and not like an outsider or something.* (Participant 8)

Similarly, one participant shared how her team positively impacted her transition,

*We have a really great team. They're really collaborative. We all share so the work gets done more efficiently, and the students get the same message and the same PowerPoints. It’s not like one gets more than the other, which is really important because there's a lot of moving parts.* (Participant 9)

Socialization with senior colleagues, co-teachers, and peers provided valuable support to the novice faculty members. When socialization occurred, the participants expressed that their transition was easier; when it was missing the participants expressed frustration in wasting time and energy navigating the transition alone.

## Discussion

### Emotional Aspects of the Transition Experience

In this study, participants experienced a range of emotions, both negative and positive feelings, during their transition experiences. Many of the participants were unfamiliar with the expectations and responsibilities associated with their new role which resulted in negative emotions. These included feeling overwhelmed and anxious, shocked, and surprised, and alone. In the nursing literature, these along with other negative emotions (i.e. frustration and burnout) have been expressed by novice nursing faculty (Fritz, 2018; Grassley et al., 2020; Jeffers & Mariani, 2017; Poorman & Mastorovich, 2017; Ross & Kerrigan, 2020). Negative emotions may impact the transition experience leading to decreased job satisfaction and an increased intent to leave for novice nurses (Miner, 2019).

The literature often highlights the challenges and negative emotions experienced during professional transitions, with limited research related to the positive aspects or emotions experienced. The participants shared that they were optimistic about their new role, and although the experience was challenging, they were able to find the positives. Similar to the qualitative study by Miner (2019) that identified positive aspects of the transition to nursing academia and the integrative review by Wendler and colleagues (2021) aimed at describing the needs of novice nursing faculty, feeling valued or having a sense of belonging within the nursing faculty increased the positive emotions of the experience and supported a successful transition.

#### Impact of COVID-19

A unique finding of the current study was the additional stress of learning to navigate the institutional virtual platforms and online communities, especially during the COVID-19 pandemic. Institutions often modified policies and procedures, and the participants shared that they needed flexibility. There is limited research examining how the abrupt shift to online learning during the pandemic impacted nursing faculty. However, in a cross-sectional international study (30 participating nursing schools from 30 countries) by Kalanlar (2022), 65% of nursing educators believed they were unprepared to switch to online education during the pandemic. Similar to the recommendation of dedicated technology support by the current study participants, Kalanlar (2022) found that the most common suggestion for the development of nursing education during the pandemic was providing educators with technical support and training on distance education technologies.

Additionally, the COVID-19 pandemic may have exacerbated isolation as all the participants shared that they often work from home. Without the interaction with colleagues, participants shared they were unsure whether they were meeting expectations and missed the collaboration often present in team nursing. Participants sought reassurance about their performance as professors, and the lack of this feedback increased their stress and made them question their abilities. Thompson and Christian (2022) examined the psychological impact of the pandemic on academics and found that without additional institutional support, faculty reported increased feelings of isolation, panic, and anxiety. The hybrid work environment of the current study participants decreased their opportunities to connect with other faculty members on campus, potentially decreasing their senses of belonging and increasing their feelings of isolation.

### Preparation for the Nursing Professor Role

The participants had various nursing graduate educations that included different coursework and teaching opportunities. Traditionally, Master’s-level graduate nursing programs do not prioritize the preparation of professors. Instead, their focus tends to lie in clinical practice and equipping nurses with the skills necessary for formal leadership roles, health management or advanced clinical skills. The participants shared that a lack of practical teaching opportunities and limited feedback from other professors on their teaching abilities were the main barriers to feeling prepared to teach. The participants expressed the need for additional education on handling student issues and mental health, as well as providing, and managing feedback. They also highlighted the importance of developing appropriate evaluation measures, stating that such training would be beneficial. Studies have shown that a lack of teaching preparation impacts nursing faculty recruitment, job satisfaction and retention (Brower et al., 2022; Jeffers & Mariania, 2017; Nicholls & Kelman, 2023; Summers, 2017; Wendler et al., 2021). The research suggests that adequate pedagogical preparation decreases frustration and stress for novice nursing faculty and improves their transition experiences (Fritz, 2018; Garner & Bedford, 2021; Laari et al., 2023).

Additionally, study participants highlighted that there is a lack of consistency across orientation programs, impacting their preparation for the professor role. Orientation programs varied in the delivery models; some were delivered in-person or virtually and others consisted of self-directed components. The participants also expressed a need for a nursing faculty-specific orientation program. They felt that a nursing faculty orientation would better address their knowledge gaps and help them prepare students for the NCLEX, a unique professional exam for nursing students. Wendler et al. (2021) found that structured orientations contributed to successful transitions to the faculty role and were crucial to faculty retention. Furthermore, nursing faculty-specific orientation program would fill the gaps in knowledge by providing structured support, training, and education to novice nursing professors (Young-Brice et al., 2022).

One contributor to preparation for the nursing professor role was the participant’s realization that they had many transferable skills from clinical practice, such as time management, communication, and collaboration. Several articles exploring the transition from expert nurse to novice educator suggested that a common facilitator for the transition was using skills from previous workplace settings (Fritz, 2018; Harper-McDonald & Taylor, 2020). These skills included leadership, intra and interprofessional communication, teamwork, program evaluation, clinical expertise, and organization. Fox (2017) found that novice nursing professors established credibility, gained self-confidence and validated their competency when they reflected on their transferable clinical expertise.

The study interviews revealed that all nine of the participants continued to work clinically in addition to their faculty role. Maintaining a clinical position was unexpected due to the workload demands of nursing faculty described in the literature and within the participant interviews (Boamah et al., 2021; Fritz, 2018; Grassley et al., 2020). Participants shared that they wanted to maintain their clinical practice to remain credible and to bring real-world experiences into the classroom. Smith and Boyd (2012) reported similar findings in their study of healthcare academics, noting that healthcare practitioners hold onto their clinical identities rather than quickly adopting the new identity of scholar or researcher. The authors further explored this concept, suggesting that professors, particularly in nursing, feel a strong responsibility to maintain their clinical credibility and to prepare students for professional life beyond academia (Boyd & Smith, 2016). Similarly, Baldwin et al. (2017) use the term *clinical currency* to describe the confidence and clinical presence nursing academics need to keep up to date with evidence-based practice and to reconcile their professional identities. It is unknown whether maintaining the clinical role positively or negatively impacted the participants’ transition experiences, as this was not directly addressed in this study. Future qualitative studies exploring this phenomenon could build on the knowledge gained from this study.

### Navigating the Role and College Setting

A significant finding of this study was the crucial role ongoing support and connections with other faculty members played in supporting novice college professors in navigating their new roles and settings. When the current study participants were supported through socialization and welcomed into the college environment, they spoke positively about building relationships and developing camaraderie with their more experienced colleagues. Effective socialization brings about several anticipated outcomes, such as the adoption of cultural norms, values, and standards, role integration, and a sense of belonging to a community (Masters & Gilmore, 2021; Yanik & Yildiz, 2019). For nursing professors, socialization is an intentionally supportive relationship between an experienced faculty member and a novice faculty member and aims to prepare and integrate new professors into the faculty group (Nicholls & Kelman, 2023). Without community support, novice nursing professors expressed decreased job satisfaction, frustration, burnout, and an increased intent to leave their position (Boamah et al., 2021; Jeffers & Mariani, 2017; Poorman & Mastorovich, 2017; Ross & Kerrigan, 2020).

### Recommendations

A significant finding was that socialization positively impacted transition experiences. A strategy to increase socialization is to create opportunities to develop relationships with experienced faculty, such as through co-teaching, collaborating on projects, and offering networking opportunities. Moreover, socialization includes activities that increase belonging to the faculty group such as administrators formally recognizing the strengths of their professors, promoting civility in the department, and offering opportunities for professional development (Nicholls & Kelman, 2023; Singh et al., 2022). Academic leaders can promote civility amongst faculty, through modeling respectful communication, providing encouragement and feedback, and through fostering purposeful relationships between experienced and novice staff (Clark, 2017).

For nurses interested in pursuing teaching roles, the study findings suggest that more practical teaching experience would benefit graduate nursing students hoping to teach within BScN programs. Graduate nursing programs could offer more courses focused on curriculum design and evaluation, online teaching, nursing pedagogy, and adult learning principles to better prepare novice faculty for their roles. Additionally, partnering with the faculty of education and providing nurses with a post-graduate teaching certification might also be helpful.

This study’s findings have significant implications for nursing leaders and administrators at nursing schools in Ontario and beyond. A key finding of this study was that nursing professors at Ontario colleges felt unprepared for their role and unanimously expressed a need for nursing-specific orientations. A comprehensive nursing faculty orientation would include an overview of the institution’s policies and procedures, preparation in nursing pedagogy, and ongoing support through mentorship and socialization. The insights gained through this research can serve as a building block to developing a provincial nursing faculty orientation program aimed at improving the transition experiences of nursing faculty and increasing retention of this valuable resource.

Finally, this study provides insight into nurse faculty transition experiences and normalizes the emotions experienced by novice nursing professors. By highlighting the transition experiences of novice nursing professors, the study findings can inform and prepare clinical nurses interested in pursuing faculty positions, as well as novice faculty transitioning into their roles.

### Limitations

The study had limitations due to its small sample size and the homogeneity of the participants, who were all women nursing professors, and therefore, other gender perspectives were not explored. Additionally, the wide age range of participants indicates diverse life experiences, which could influence their descriptions. Demographic information related to race was not collected, however, it is well known that Black, Indigenous, and People of Color continue to be underrepresented in nursing graduate programs and leadership positions in Canada (Nelson & Salami, 2021). Future studies should focus on exploring the experiences of minority faculty members to develop tailored recruitment and retention strategies in academia. Increasing diversity in nursing academic leadership is essential for the sustainability of the nursing profession as it more accurately reflects the diversity of Canada (Nelson & Salami, 2021).

## Conclusion

This study is among the first to explore the experiences of nurses transitioning from clinician to professor at Ontario colleges, particularly during a global pandemic, highlighting both the challenges and supports required to aid in their successful transition. The transition experience is described as an emotional journey with positive and negative emotions experienced over time. Despite their new roles as professors, participants remained connected to their clinical identities, using their professional experiences to navigate their new roles effectively. All participants felt a strong responsibility to provide an excellent education to the next generation of nurses. Previous literature emphasizes the importance of structured orientations and mentorships for novice faculty, which helps them develop their teaching skills and become confident in their roles. The findings of this study support the need for evidence-informed orientation programs that help navigate the academic culture of degree nursing programs. Finally, the study provides new insights into novice nursing faculty members’ socialization experiences, underscoring the crucial role socialization plays in a successful transition experience.
